# Distal Limb Ischaemia in Association With Takotsubo Cardiomyopathy

**DOI:** 10.7759/cureus.43301

**Published:** 2023-08-10

**Authors:** Keng Han Yeap, Charles Badu-Boateng, Mark Lloyd

**Affiliations:** 1 Cardiology, London North West University Healthcare National Health Service (NHS) Trust, London, GBR; 2 Cardiology, Royal Berkshire National Health Service (NHS) Foundation Trust, Reading, GBR; 3 Rheumatology, Frimley Health National Health Service (NHS) Foundation Trust, Camberley, GBR

**Keywords:** takotsubo syndrome, raynaud’s phenomenon, myocardial stunning, catecholamines excess, distal limb ischaemia, takotsubo cardiomyopathy

## Abstract

Raynaud’s phenomenon (RP) is a common clinical condition associated with digital ischaemia. A 73-year-old woman with a history of RP presented with bilateral distal lower limb ischaemia. Although no chest pain was reported, her serum troponin was greater than 25,000 ng/ml with lateral lead ST-segment elevation on ECG. Her coronary angiogram was normal, but echocardiography revealed a hypokinetic apical region consistent with Takotsubo cardiomyopathy. She was treated with iloprost, but her toes became necrotic, mummified and auto-amputated over six months. It is hypothesised that a surge in serum catecholamines may link the two processes.

## Introduction

Raynaud’s phenomenon (RP) has a prevalence of approximately 5% and is characterised by spasm of digital vasculature, leading to a reduction in blood supply to the digits [1-3]. It is episodic and there is often an identifiable trigger such as stress or low environmental temperature [1]. It can be idiopathic or secondary to other conditions such as systemic sclerosis [1]. RP may be benign and managed with conservative or pharmacological measures, but it can also be refractory and cause severe digital ischaemia and necrosis [1].

Takotsubo cardiomyopathy is a rare condition that mainly affects post-menopausal women [4]. It was first described in 1991 and is characterised by transient left ventricular (LV) apical dilatation and dysfunction [4,5]. Its clinical presentation may mimic acute coronary syndrome (ACS) as patients present with chest pain, ischaemic ECG pattern and positive serum troponin in the absence of significant coronary artery occlusion [4]. The majority of cases recover fully within weeks but approximately one-fifth develop acute cardiac complications such as acute heart failure or cardiogenic shock [4].

This case was initially presented as a poster at the 2022 British Society for Rheumatology (BSR) Annual Conference held in Glasgow, United Kingdom, from 25 to 27 April 2022, and an abstract was published in the Rheumatology Journal supplement in May 2022 [6].

## Case presentation

A 72-year-old female experienced persistent pain and discomfort in both her feet associated with a change in colour for five days. She did not have chest pain or dyspnoea [6].

She had a history of hypertension which was treated with ramipril and RP with previous similar digital symptoms which were less severe and resolved spontaneously. She had a history of alcohol misuse and had recently been admitted with an alcohol withdrawal seizure. She was a non-smoker and otherwise independent in relation to her daily living activities with no requirement for walking aids. She also experienced several recent stressful life events [6].

**Figure 1:**
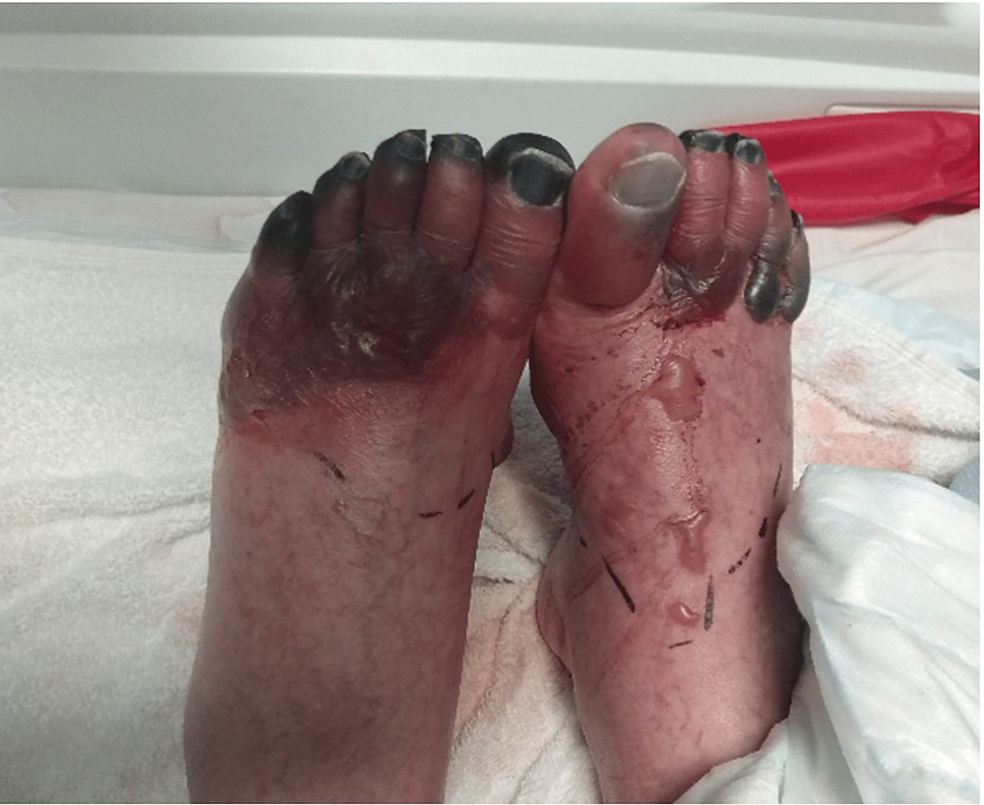
Dark and dusky distal feet more pronounced on the left foot

On clinical examination, her toes were dusky blue, and her distal feet were tender with diminished touch sensation overlying them (Figure 1). The proximal feet were warm on palpation. Visual examination of the fingernail fold was normal with no obvious capillary enlargement, haemorrhages and fingernail pitting. Her blood pressure on admission was 110/78, and peripheral vascular examination noted palpable pedal pulses bilaterally with good biphasic signal from an ultrasound Doppler scan. Her heart sound was normal and the rest of the physical examination was unremarkable [6].

**Figure 2:**
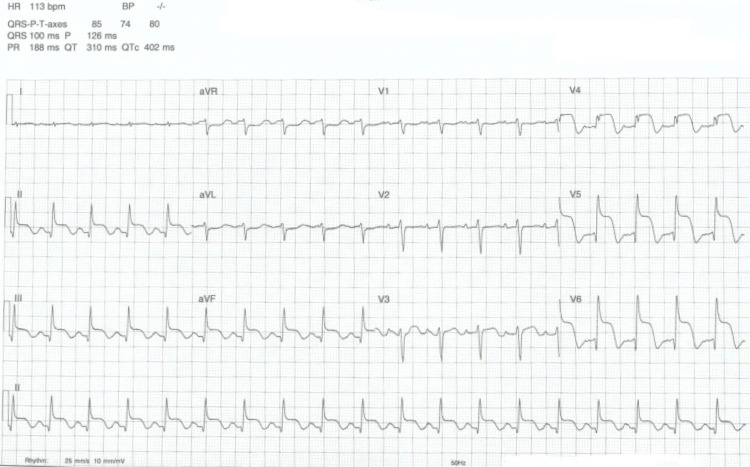
ECG showing ST-segment elevation in the inferolateral leads

**Figure 3:**
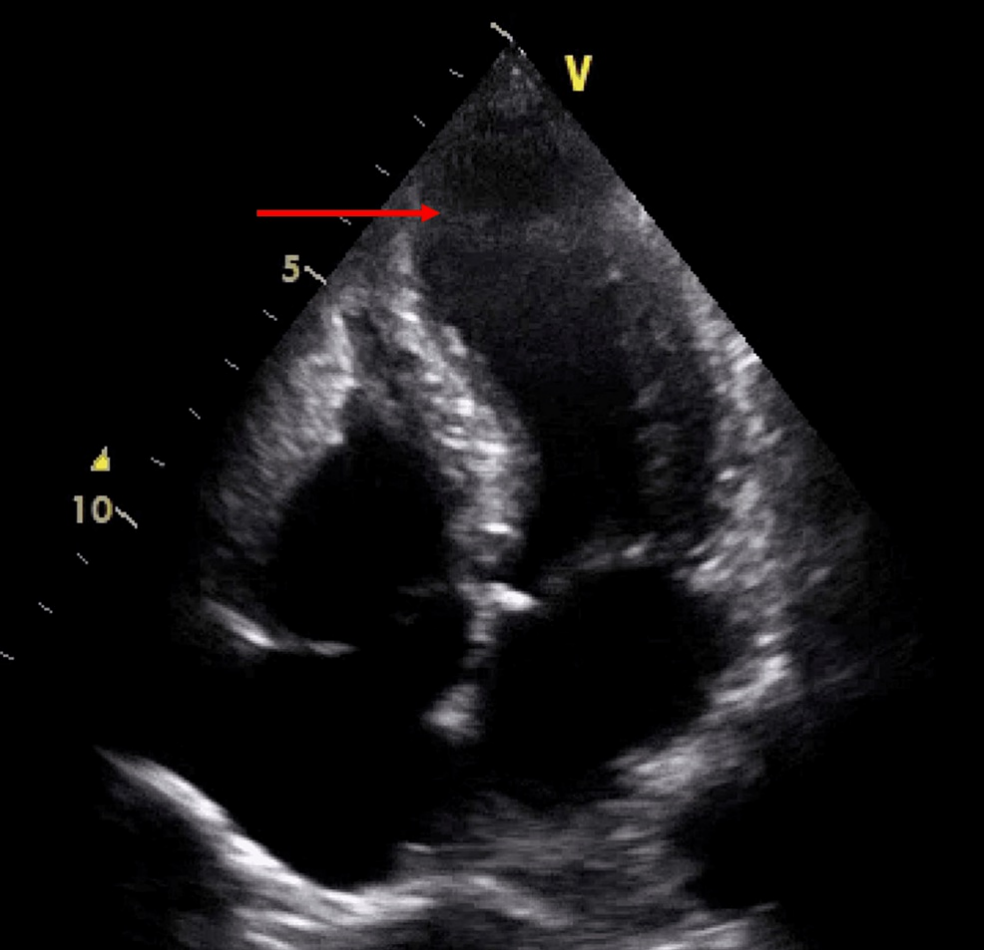
Apical four-chamber view of echocardiography showing apical hypokinesia and ballooning during ventricular systole

Her ECG showed ST-segment elevation in the inferolateral leads (Figure 2), and serum troponin was greater than 25,000 ng/ml. Thus, she was initially treated for ACS with dual antiplatelet therapy and received intravenous heparin infusion in view of the possibility of lower limb thrombotic disease. However, coronary angiography subsequently revealed normal coronary vessels, and the echocardiogram showed a hypokinetic apex (Figure 3) with an estimated LV ejection fraction of 35-40%, in keeping with Takotsubo cardiomyopathy [6].

During the admission, the toes on both her feet displayed signs of worsening ischaemia. An ultrasound scan of the lower limb venous system showed a chronic thrombus in the left long saphenous vein, but the rest of the superficial venous system was patent and competent. No evidence of pulmonary embolism or right heart strain was noted on her computerised tomography pulmonary angiography, apart from incidental bi-basal pleural effusions and lower lobe atelectasis. Her white cell count and C-reactive protein were noted to be elevated, and broad-spectrum antibiotics were commenced [6].

Urine catecholamines were measured on the eighth day of admission, and normetadrenaline, metadrenaline and 3-methoxytyramine levels were normal. Rheumatoid factor, anti-nuclear antibody, anti-neutrophil cytoplasmic antibody, double-stranded DNA antibody and serum cryoglobulins, hepatitis B and C serology were all negative [6].

Outcome and follow-up

An infusion of iloprost was administered daily over five days, and this resulted in a gradual resolution of the pain in her feet. However, the appearance and colour of the distal feet remained unchanged with necrotic toes. Her mobility improved with physiotherapy assistance, and she was subsequently discharged from the hospital on aspirin, furosemide, spironolactone and ivabradine for her cardiac dysfunction. Anticoagulation therapy was discontinued as there was no evidence of acute venous thromboembolism [6].

**Figure 4:**
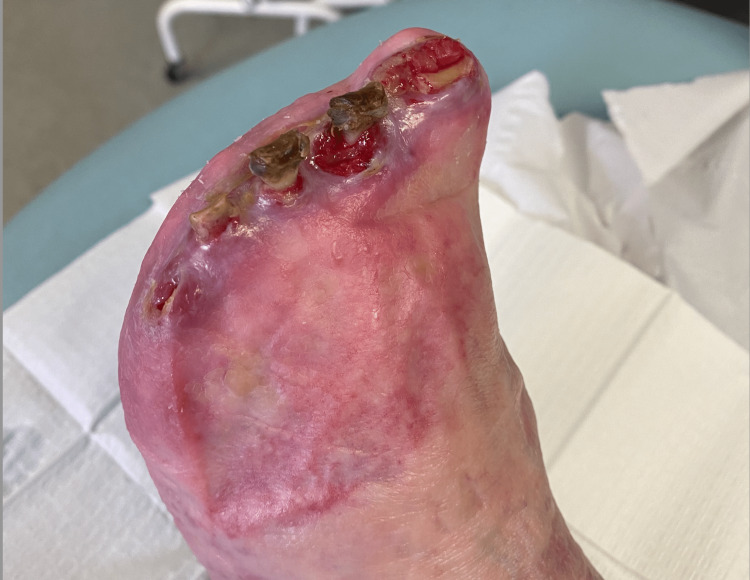
Left foot after auto-amputation of all toes

Throughout her follow-up appointments over six months, mummification and eventually auto-amputation of her toes ensued (Figure 4). No further digital ischaemia was reported and she remained well otherwise [6].

## Discussion

The pathophysiology of Takotsubo cardiomyopathy is not fully understood. A well-accepted hypothesis is that a surge in serum catecholamines may lead to a spasm of cardiac microvasculature as well as stunning of myocardium [4]. However, catecholamines have a very short half-life of one to two minutes, posing challenges in clinical diagnosis and investigation [7]. In this case, urine catecholamines were measured several days after the initial acute presentation in an attempt to exclude phaeochromocytoma and these were within normal limits.

Takotsubo cardiomyopathy has been observed in the context of RP [8]. However, patients with RP appear to have normal levels of endogenous catecholamines [9]. A dusky hand appearance suggestive of RP with associated ischaemia of the digits was previously reported in the context of phaeochromocytoma, and the appearance improved following the removal of the tumour [10].

The temporal relationship between Takotsubo cardiomyopathy and ischaemic toes in this patient suggests a shared pathology. However, to the authors’ knowledge, there has not been any previous case report of digital ischaemia in association with Takotsubo cardiomyopathy. It is possible that a combination of a surge in catecholamines in Takotsubo cardiomyopathy on a background of RP caused the critical toe ischaemia observed in this case. The prevalence of RP is high amongst the general population and in view of the significant impact of the postulated surge in serum catecholamines on the myocardium in Takotsubo cardiomyopathy, the fact that the associated digital ischaemia and ischaemia of other organ systems is uncommonly observed is surprising [6].

## Conclusions

The pathophysiology of Takotsubo cardiomyopathy remains poorly understood, and there is no reliable test for early diagnosis of Takotsubo cardiomyopathy. Takotsubo cardiomyopathy should be considered in patients presenting with acute distal limb ischaemia upon exclusion of peripheral vascular disease. This is because early identification of Takotsubo cardiomyopathy and prompt supportive treatment can improve long-term outcomes. Catecholamines have a short serum half-life, and serum samples should ideally be obtained when Takotsubo cardiomyopathy is suspected.
